# Detoxification of Reactive Carbonyl Species by Glutathione Transferase Tau Isozymes

**DOI:** 10.3389/fpls.2019.00487

**Published:** 2019-04-24

**Authors:** Jun’ichi Mano, Sayaka Kanameda, Rika Kuramitsu, Nagisa Matsuura, Yasuo Yamauchi

**Affiliations:** ^1^Science Research Center, Organization for Research Initiatives, Yamaguchi University, Yamaguchi, Japan; ^2^Faculty of Agriculture, Yamaguchi University, Yamaguchi, Japan; ^3^Graduate School of Agricultural Science Kobe University, Kobe, Japan

**Keywords:** acrolein, lipid peroxide, oxidative stress, oxylipin, reactive electrophile species, redox signal

## Abstract

Oxidative stimuli to living cells results in the formation of lipid peroxides, from which various aldehydes and ketones (oxylipin carbonyls) are inevitably produced. Among the oxylipin carbonyls, those with an α,β-unsaturated bond are designated as reactive carbonyl species (RCS) because they have high electrophilicity and biological activity. Plants have arrays of dehydrogenases and reductases to metabolize a variety of RCS that occur in the cells, but these enzymes are not efficient to scavenge the most toxic RCS (i.e., acrolein) because they have only low affinity. Two glutathione transferase (GST) isozymes belonging to the plant-specific Tau class were recently observed to scavenge acrolein with *K*_M_ values at a submillimolar level. This suggests that GST could also be involved in the defense system against RCS. We tested the activities of 23 Tau isozymes of *Arabidopsis thaliana* for five types of RCS, and the results revealed that 11 isozymes recognized either acrolein or 4-hydroxy-(*E*)-2-nonenal or both as a substrate(s). Such RCS-scavenging activities indicate the potential contribution of GST to RCS scavenging in plants, and they may account for the stress tolerance conferred by several Tau isozymes. RCS are therefore a strong candidate for endogenous substrates of plant GSTs.

## Main Text

### Reactive Carbonyl Species (RCS) Are Signaling/Damaging Agents That Act Downstream of ROS

The production of reactive oxygen species (ROS) such as superoxide radical, hydrogen peroxide (H_2_O_2_), and singlet oxygen (^1^O_2_) intrinsically accompanies aerobic life. One important aspect of ROS *in vivo*, although not always noticed, is that ROS are often produced in the close vicinity of membranes, in association with chloroplastic and mitochondrial electron transport chains and the plasma membrane-bound respiratory burst NADPH oxidase homologs (RBOHs). Membrane lipids are therefore constitutively oxidized due to the basal generation of ROS (Mène-Saffrané et al., 2007). The resulting lipid peroxides are relatively unstable and decompose or are metabolized to a variety of compounds called oxylipins, in which many types of aldehydes and ketones (oxylipin carbonyls) with different carbon chain lengths and extents of unsaturation are present. Carbonyl compounds are more reactive than corresponding alcohols and carboxylic acids, and the α,β-unsaturated carbonyls [reactive carbonyl species (RCS)] in particular have high electrophilicity and play critical biological roles in a range of functions from gene regulation to cytotoxicity (Esterbauer et al., 1991; Farmer and Mueller, 2013). Typical and well-studied RCS are acrolein, 4-hydroxy-(*E*)-2-nonenal (HNE), 4-oxo-(*E*)-2-nonenal, and malondialdehyde (MDA). The participation of RCS in oxidative injury and oxidative signaling in cells has been established for animals (Schopfer et al., 2011).

Reactive carbonyl species, e.g., acrolein and HNE, exhibit toxicity to plant cells and organelles when they are added exogenously (Millar and Leaver, 2000; Alméras et al., 2003; Mano et al., 2005, 2009). The occurrence of RCS in plant tissues was verified by extensive carbonyl analyses as follows. Yin et al. (2010) showed that tobacco roots contain dozens of carbonyls including several RCS, and they reported that the levels of some carbonyls were increased by the toxic level of aluminum ion. In leaves also, various carbonyls have been detected in tobacco (Mano et al., 2010), *Arabidopsis thaliana* (Yamauchi et al., 2012), and cyclamen (Kai et al., 2012), and their levels were increased by a high intensity of light (Mano et al., 2010), methyl viologen (Yamauchi et al., 2012), high salinity (Mano et al., 2014), injury (Matsui et al., 2012), and heat stress (Kai et al., 2012). Table 1 summarizes the stress-related RCS and carbonyls identified in plants. The observed increases of RCS are ascribed to the increased levels of ROS by the stressors. These endogenously produced RCS were concluded to be responsible for the tissue damage because the extent of damage and the RCS levels correlated positively in transgenic plants that overexpress or lack an RCS-detoxifying enzyme (Mano et al., 2010; Yin et al., 2010; Yamauchi et al., 2012).

**Table 1:** RCS and related carbonyls that are present in plants, and the plant enzymes that metabolize the carbonyls.

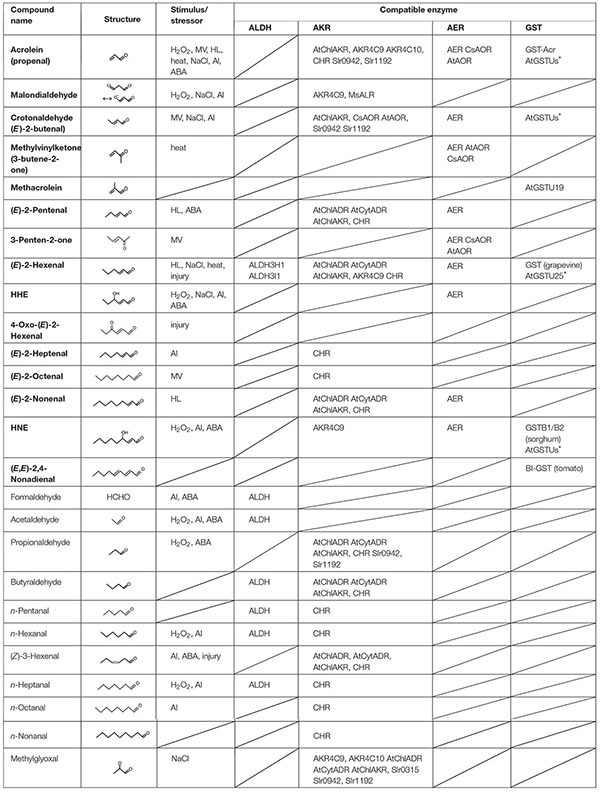

*^∗^This study. Compound names in boldface are an RCS. ’Stimulus/stressor’ indicates the exogenous stressors and endogenous stimuli reported to increase the level of each carbonyl in plants (see the main text). Compatible enzymes represent isozymes for which a catalytic activity for the corresponding carbonyl has been reported. References for enzyme activity: AER (Mano et al., 2002, 2005), AKR4C9 (Simpson et al., 2009; Saito et al., 2013), AKR4C10 (Saito et al., 2013), ALDH (Asker and Davies, 1985), ALDH3H1 and ALDH3I1 (Stiti et al., 2011), AtAOR, AtChlAKR, AtChlADR, and AtCytADR (Yamauchi et al., 2011), AtGSTU19 (Mano et al., 2017), BI-GST (Kobayashi et al., 2011), CHR (Tanaka et al., 2018), MsALR (Oberschall et al., 2000), CsAOR (Yamauchi et al., 2011), GST-Acr (Mano et al., 2017), GST B1/B2 (Gronwald and Plaisance, 1998), GST from grapevine (Kobayashi et al., 2011), and Slr0545, Slr0942, Slr1192 and Slr1503 (Shimakawa et al., 2013). Slashed boxes, No data is available*.

Reactive carbonyl species in plants also play signaling roles. Bate and Rothstein (1998) demonstrated that the exogenous addition of (*E*)-2-hexenal to *A. thaliana* plants induced a group of genes involved in defense against pathogens. The induced gene members vary by the RCS type (Alméras et al., 2003; Weber et al., 2004). Yamauchi et al. (2015) found that 2-alkenals of carbon chain length 4–8, when added as volatiles, induced heat-shock response genes in *A. thaliana*. Endogenous RCS produced upon an oxidative stimulus act as initiators of programmed cell death (PCD) in tobacco cultured cells (Biswas and Mano, 2015) by activating caspase-3-like protease (Biswas and Mano, 2016). In the stomata closure signaling of abscisic acid (ABA), the ROS production in guard cells is followed by increases in RCS, and the genetic suppression of RCS inhibited the stomata response to ABA (Islam et al., 2016). Together these observations, compiled over the past decade, indicate that RCS are endogenous agents that mediate ROS stimuli to downstream responses.

### Enzymatic Regulation of RCS

Dozens of oxylipin carbonyls in plants (Table 1) have a broad range of reactivity and thus different toxicity and signaling effects (Mano et al., 2009; Biswas and Mano, 2015). Plants have three types of oxidoreductases for metabolizing carbonyls: (i) aldehyde dehydrogenase (ALDH) to oxidize an aldehyde to a carboxylic acid with NAD^+^, (ii) aldehyde reductase to reduce an aldehyde or a ketone to a corresponding alcohol with NAD(P)H; two types of proteins, one belonging to aldo-keto reductase (AKR) family and the other to short-chain dehydrogenase/reductase family, can catalyze this reaction (Yamauchi et al., 2011), and (iii) 2-alkenal reductase (AER) and alkenal/one oxidoreductase (AOR) to reduce an RCS at the carbonyl-conjugated C-C double bond with NAD(P)H (Mano et al., 2002, 2005; Yamauchi et al., 2011). These enzyme classes, respectively, have multiple isozymes, and each isozyme shows distinct substrate specificity. Table 1 summarizes the plant isozymes of these enzyme classes and reported substrates. Some of these isozymes have been shown to detoxify carbonyls *in planta*; their overexpression in transgenic plants reduced the carbonyl levels and conferred tolerance against several types of environmental stressors (reviewed by Mano, 2012).

### Acrolein Is Scavenged by Glutathione Transferase

Among the RCS, acrolein (or 2-propenal), the C3 alkenal, is the most highly reactive and toxic compound (Esterbauer et al., 1991). It can inactivate photosynthetic machinery (Mano et al., 2009; Shimakawa et al., 2013) and induce PCD (Biswas and Mano, 2016). As seen in Table 1, many reductases recognize acrolein as a substrate, but they have higher affinity to longer-chain aldehydes and show only low affinity to acrolein, i.e., *K*_M_ values > 2 mM (Mano et al., 2017).

Acrolein reacts with the reduced form of glutathione (GSH) very rapidly (Esterbauer et al., 1975), and certain isozymes of glutathione transferase (GST) can catalyze the conjugation of acrolein with GSH. Human GST isozymes Alpha1, Mu1, and Pi1 recognize acrolein as a substrate (Berhane et al., 1994). Several plant GSTs have been known to react with RCS; for example, GST B1/B2 from sorghum recognizes HNE as a substrate (Gronwald and Plaisance, 1998). BI-GST and four Tau class isozymes from tomato reacted with (*E*,*E*)-2,4-nonadienal, and so did two isozymes from grapevine with (*E*)-2-hexenal (Kobayashi et al., 2011). We have investigated acrolein-scavenging GST activity and detected it in *A. thaliana*, spinach, rice, and Chinese cabbage. We then isolated a Tau isozyme from spinach for scavenging acrolein with the *K*_M_ value 93 μM for acrolein. A homologous GST isozyme Tau19 from *A. thaliana* (AtGSTU19) also scavenged acrolein with the *K*_M_ value 30 μM (Mano et al., 2017). The enzymatic scavenging of acrolein in plants had previously been attributed only to AER and AKR reactions (Table 1), but these GST Tau (GSTU) isozymes appear to be physiologically more relevant because their *K*_M_ values are close to the physiological range of acrolein.

### Eleven AtGSTU Isozymes Recognize RCS as Substrates

**FIGURE 1:**
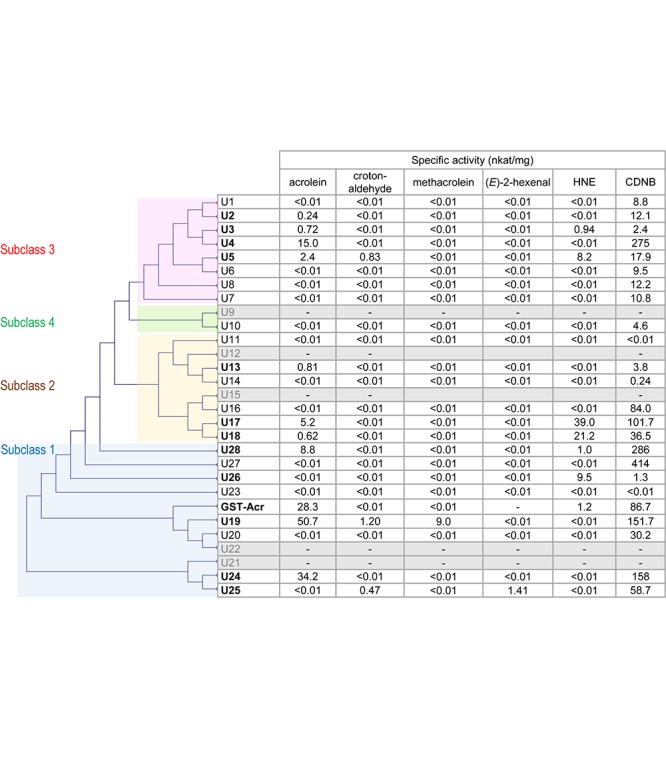
Substrate specificity of AtGSTU isozymes and spinach GST-Acr. Gray rows represent the isozymes that were not recovered as soluble protein. The assay conditions are described in the “Materials and Methods” section. The GST-Acr and AtGSTU19 data are from our earlier study (Mano et al., 2017). The isozymes are arranged in the order of the phylogenetic tree, which was constructed on the amino acid sequence similarity by the neighbor-joining method using the multiple sequence alignment software Clustal W 2.0 (Larkin et al., 2007). The amino acid sequence of the spinach GST-Acr was deduced from the assembled RNA sequence (Mano et al., 2017).

GST Tau is a plant-specific class and is the largest group of GST isozymes in angiosperms, gymnosperms, and ferns (Flova, 2003; Monticolo et al., 2017). GSTU isozymes have important roles in plants’ defense against environmental stress. The overexpression of a GSTU gene from the extreme halophyte *Salicornia brachiata* (Jha et al., 2011) in tobacco conferred salt tolerance. *A. thaliana* plants overexpressing rice *OsGSTU4* gene (Sharma et al., 2014), *AtGSTU17* gene (Chen et al., 2012), and *AtGSTU19* gene (Xu et al., 2016) showed more tolerance to salinity and oxidative stress than the wild type. GSTU isozymes therefore constitute part of the anti-oxidative defense, but the underlying biochemical mechanism remains unclear because the physiologically relevant substrates of GSTU have not been elucidated. The efficient acrolein-scavenging activity observed in the two GSTU isozymes described above suggested to us the possibility that GSTU isozymes can be counted as RCS-scavenging enzymes. To test this possibility, we determined the RCS-scavenging activity of AtGSTU isozymes (Figure 1).

Complementary DNA of 28 AtGSTU isogenes was cloned and expressed in *Escherichia coli*, and pure recombinant proteins were obtained (see Materials and Methods). Recombinant AtGSTU9, AtGSTU12, AtGSTU15, AtGSTU21, and AtGSTU22 proteins were not recovered as the soluble form. The isozymes obtained as soluble proteins (23 in total) were first tested for activity for a universal GST substrate 1-chloro-2,4-dinitrobenzene (CDNB). Recombinant AtGSTU11 and AtGSTU23 proteins were incompetent, and the other 21 showed the CDNB-conjugating activity.

We then examined the 23 isozymes for the activity to scavenge five types of RCS, i.e., acrolein, crotonaldehyde, methacrolein, (*E*)-2-hexenal, and HNE (Figure 1). Acrolein-scavenging activity was detected (higher than 0.01 nkat mg^-1^) in the following ten isozymes: AtGSTU2, AtGSTU3, AtGSTU4, AtGSTU5, AtGSTU13, AtGSTU17, AtGSTU18, AtGSTU19, AtGSTU24, and AtGSTU28. The specific activity of these isozymes ranged from 0.24 nkat mg^-1^ (AtGSTU2) to 50.7 nkat mg^-1^ (AtGSTU19). These results show that acrolein is a common endogenous substrate of GSTU.

For crotonaldehyde, three isozymes (AtGSTU5, AtGSTU19, and AtGSTU25) showed significant activity. For methacrolein, only AtGSU19 and for (*E*)-2-hexenal only AtGSTU25 showed the activity. For HNE, six isozymes (AtGSTU3, AtGSTU5, AtGST17, AtGSTU18, AtGSTU26, and ATGSTU28) exhibited significant activity. In total, at least 11 of the 28 isozymes showed RCS-scavenging activity.

GST Tau isozymes are grouped into four subclasses based on their amino acid sequence similarity (Monticolo et al., 2017) as indicated in Figure 1. RCS-scavenging activity was identified in the isozymes in subclasses 1, 2, and 3, and these three subclasses also have RCS-incompatible isozymes (Figure 1). This suggests that the acquisition of the RCS-recognizing ability during the molecular evolution of AtGSTUs occurred multiple times independently. An alternative possibility is that the common ancestor of AtGSTU had the RCS-scavenging activity and it was lost during the molecular evolution.

### The Physiological Relevance of the RCS-Scavenging Activity of GST

Among these 11 RCS-compatible isozymes, AtGSTU13 and AtGSTU19 are expressed in almost all tissues except the male organ (Supplementary Table S2, data extracted from Dixon et al., 2010). In shoot tissues, AtGSTU1, AtGSTU17, and AtGSTU18 are strongly expressed, and in root tissues, AtGSTU2, AtGSTU4, AtGSTU24, and AtGSTU28 are expressed. AtGSTU18 and AtGSTU19 are constitutively expressed. In particular, AtGSTU19 is the most abundant GSTU isozyme in *A. thaliana* (Dixon and Edwards, 2009) and appears to be a key isozyme to protect the whole plant body from the toxicity of RCS, especially acrolein. Other isozymes, in contrast, are induced by various stressors such as salt, high osmolarity, and UV-B [Supplementary Table S2, from AtGenExpress database (Kilian et al., 2007)], which commonly increase the intracellular levels of ROS. Notably, two isozymes (AtGSTU17 and AtGSTU19) show relatively high RCS-scavenging activities, and they conferred stress tolerance to transgenic plants (Chen et al., 2012; Xu et al., 2016). The physiological function of GSTUs as RCS scavengers can be verified by analyses of the RCS levels in these samples.

We reported the acrolein-scavenging GST activity in leaf extracts of *A. thaliana*, *Brassica rapa* var. *pekinensis* (both are Brassicales), *Oryza sativa* (Poales), and *Spinacia oleracea* (Caryophyllales), ranging from 120 to 255 nmol/min/mg protein, as determined by the HPLC analysis of the acrolein decrease rate (Mano et al., 2017). We here detected the activity in four more species (values indicate the activity in nmol/min/mg protein): extracts from leaves of *Allium cepa* (Liliales), 185; *Apium graveolens* var. *dulce* (Apiales), 148; *Glebionis coronaria* (Asterales), 208, and green bell fruits of *Capsicum annuum* var. *grossum* (Solanales), 155. The occurrence of the activity in all tested species (seven orders of angiosperms) supports the importance of acrolein-scavenging GST activity in plants.

### Conclusion

It was revealed that at least 11 of the 28 GSTU isozymes in *A. thaliana* can recognize RCS as substrates, indicating that RCS are important endogenous substrates of GSTU. Some members of the RCS-compatible GSTU isozymes are expressed in various tissues constitutively, and others are induced by a variety of environmental stressors. Acrolein-scavenging GST activity was observed in a broad range of angiosperms at similar levels of specific activity. These findings demonstrate that RCS-scavenging GST activity is a significant element constituting the anti-oxidative defense in plants.

## Materials and Methods

### The cDNA Cloning of AtGSTUs, Expression, and Purification of Recombinant Proteins

Recombinant AtGSTU19 with an *N*-terminal poly(His) tag was obtained as described (Mano et al., 2017). For other isogenes, the cDNA of the corresponding open reading frame (ORF) was obtained by polymerase chain reaction (PCR)-based cloning. Briefly, total RNA was prepared from 3-week-old *A. thaliana* using an RNeasy Plant Mini Kit (Qiagen, Hilden, Germany), and then cDNA was synthesized by a ReverTra Ace Kit (Toyobo, Osaka, Japan). The ORF of the GSTU was amplified by PCR using proof-reading KOD DNA polymerase (Toyobo) and the primers listed in Supplementary Table S1. Amplified PCR products were subcloned into pMD19 (Takara Bio, Shiga, Japan), and the identity of subcloned cDNA was verified by DNA sequencing. The confirmed ORF of the GSTU was ligated into the multicloning site in pASK15-plus expression vector (IBA, Göttingen, Germany), which produces the recombinant GSTU comprising an *N*-terminal streptavidin tag.

*Escherichia coli* BL21 cells transformed with the expression plasmid were grown at 37°C in LB broth containing 100 μg ml^-1^ ampicillin. Expression of the transgene was induced with anhydrotetracycline, and the recombinant protein was purified via affinity chromatography using Strep-Tactin Sepharose (IBA) according to the manufacturer’s instruction. The purity of the GSTU was verified by sodium dodecyl sulfate-polyacrylamide gel electrophoresis (SDS–PAGE). The purified GSTU fraction was dialyzed against 10 mM Tris–HCl, pH 7.5, and finally mixed with an equal volume of 80%(v/v) glycerol and stored at -20°C until use.

### The Assay Conditions for Each Substrate

CDNB: 100 mM potassium phosphate buffer, pH 6.5, 1.0 mM GSH, and 1.0 mM CDNB. The activity was monitored as the rate of absorbance increase at 340 nm (extinction coefficient 9.6 mM^-1^ cm^-1^). Acrolein, methacrolein, and (*E*)-2-hexenal: 10 mM MES-NaOH, pH 6.0, 0.1 mM GSH and 0.1 mM aldehyde [for (*E*)-2-hexenal, 0.05 mM]. Absorbance decreases at 215 nm (15.0 mM^-1^ cm^-1^) for acrolein, 220 nm (10.96 mM^-1^ cm^-1^) for methacrolein, and 224 nm (19.5 mM^-1^ cm^-1^) for (*E*)-2-hexenal. Crotonaldehyde and HNE: 100 mM Na-phosphate buffer, pH 7.5, 0.1 mM GSH, and 0.1 mM aldehyde. Absorbance decreases at 240 nm (10.7 mM^-1^ s^-1^) for crotonaldehyde and 221 nm (13.1 mM^-1^ cm^-1^) for HNE. The rate of non-enzymatic conjugation was subtracted as a background.

## Author Contributions

JM and YY conceived the project and wrote the manuscript with contributions from all of the authors. NM performed the overexpression and purification of proteins. SK and RK analyzed the GST activity.

## Conflict of Interest Statement

The authors declare that the research was conducted in the absence of any commercial or financial relationships that could be construed as a potential conflict of interest.
