# Supplementary material for: Detoxification of Reactive Carbonyl Species by Glutathione Transferase Tau Isozymes
Source: Front Plant Sci. 2019 Apr 24;10:487. doi: 10.3389/fpls.2019.00487 (PMC6491729; doi:10.3389/fpls.2019.00487)
Supplement: Supplementary file 1 [file Table_1.docx]

Supplementary Material

Detoxification of Reactive Carbonyl Species by Glutathione Transferase Tau Isozymes

Jun’ichi Mano*, Sayaka Kanameda, Rika Kuramitsu, Nagisa Matsuura, Yasuo Yamauchi*

*** Correspondence:** Co-corresponding Authors: mano@yamaguchi-u.ac.jp, yamauchi@kobe-u.ac.jp

# Supplementary Table 1

List of primers used for GSTU cloning. Underline indicates the digestion site with indicated restriction enzyme.

| Primer name | Sequence | Restriction enzyme |
| --- | --- | --- |
| GSTU1 5´ | ATGGTAGGTCTCACTCCATGGCGGAGAAAGAAGAGAGTGT | *BsaI* |
| 3´ | ATGGTAGGTCTCATATCAGGCAGACTTAATTGTCTCTGCAAT | *BsaI* |
| GSTU2 5´ | ATGGTAGGTCTCACTCCATGGCGAAGAAAGAAGAGAGTGTG | *BsaI* |
| 3´ | ATGGTAGGTCTCATATCAGAACGTAGACTTAGCTCTCTCTAT | *BsaI* |
| GSTU3 5´ | ATGGTAGGTCTCACTCCATGGCCGAGAAAGAAGAGGGTG | *BsaI* |
| 3´ | ATGGTAGGTCTCATATCAGACCGCTTTGATTCGTCCTACAA | *BsaI* |
| GSTU4 5´ | GGATCCATGGCGGAGAAAGAAGAGGA | *Bam*HI |
| 3´ | GTCGACTTAGGCTGATTTGATTCTTT | *Sal*I |
| GSTU5 5´ | ATGGTAGGTCTCACTCCATGGCTGAGAAAGAAGAAGTGAAG | *BsaI* |
| 3´ | ATGGTAGGTCTCATATCAAGAAGATCTCACTCTCTCTGCCA | *BsaI* |
| GSTU6 5´ | ATGGTAGGTCTCACTCCATGGGCAAAAATGAGGAAGTGAAG | *BsaI* |
| 3´ | ATGGTAGGTCTCATATCAAGCAGATCTAATTTTCTCTGCCATA | *BsaI* |
| GSTU7 5´ | ATGGTAGGTCTCACTCCATGGCGGAGAGATCAAATTCAGAG | *BsaI* |
| 3´ | ATGGTAGGTCTCATATCAAGCAGATTTGATATTGAGTTTCTCC | *BsaI* |
| GSTU8 5´ | ATGGTAGGTCTCACTCCATGAACCAAGAAGAGCACGTAAAG | *BsaI* |
| 3´ | ATGGTAGGTCTCATATCAATTAGATGTAACACTTCCAAACATGG | *BsaI* |
| GSTU10 5´ | ATGGTAGGTCTCACTCCATGGAGGAGAAGAAGAGCAAAGTG | *BsaI* |
| 3´ | ATGGTAGGTCTCATATCATGCATTTGCAGCCTGCTGGAGA | *BsaI* |
| GSTU11 5´ | ATGGTAGGTCTCACTCCATGGGTCTAATGAATCGGTCAAAG | *BsaI* |
| 3´ | ATGGTAGGTCTCATATCATTTAAAGATTGAAGTATTGAACTTGAG | *BsaI* |
| GSTU13 5´ | GAATTCatggctcagaacgatacagt | *EcoR*I |
| 3´ | GGATCCtcactgaacattgaactttt | *Bam*HI |
| GSTU14 5´ | ATGGTAGGTCTCACTCCATGGCTCAGAACGATACAGTGAAG | *BsaI* |
| 3´ | ATGGTAGGTCTCATATCATTCATATTCCGAAGTCGAAACATTG | *BsaI* |
| GSTU16 5´ | ATGGTAGGTCTCACTCCATGGGAGAGAAAGAGGAAGTGAAA | *BsaI* |
| 3’ | ATGGTAGGTCTCATATCAAGATCTTGAAGCTGCGGATTGAG | *BsaI* |
| GSTU17 5´ | ATGGTAGGTCTCACTCCATGGCAAGCAGCGACGTGAAGC | *BsaI* |
| 3´ | ATGGTAGGTCTCATATCAGGCCTGCGGCTTAGGAAAGATC | *BsaI* |
| GSTU18 5´ | GGATCCatggcgaccgaggacgtgaa | *Bam*HI |
| 3´ | GTCGACttatgcttgccgcttaggaa | *Sal*I |
| GSTU20 5´ | ATGGTAGGTCTCACTCCATGGCGAACCTACCGATTCTTTTG | *BsaI* |
| 3´ | ATGGTAGGTCTCATATCAGAGATTGTTCTTCCTATACTCAGC | *BsaI* |
| GSTU21 5´ | GGATCCATGGCAGCCGAAGTGATCCT | *Bam*HI |
| 3´ | GTAGACTTACTCAATTCCATATAACT | *Sal*I |
| GSTU23 5´ | ATGGTAGGTCTCACTCCATGGAGGAAGAGATTATCCTATTGG | *BsaI* |
| 3’ | ATGGTAGGTCTCATATCAATCGATTCCTAAAATAATCTTCCGAT | *BsaI* |
| GSTU24 5´ | GGATTCATGGCAGATGAGGTGATTCT | *Bam*HI |
| 3´ | GTCGACTTACTCCAACCCAAGTTTCT | *Sal*I |
| GSTU25 5´ | ATGGTAGGTCTCACTCCATGGCAGACGAGGTGATTCTTCT | *BsaI* |
| 3´ | ATGGTAGGTCTCATATCATTCGATTTCGATCCCAAGTTTTTTC | *BsaI* |
| GSTU26 5´ | ATGGTAGGTCTCACTCCATGGCGAACGACCAAGTGATTCT | *BsaI* |
| 3´ | ATGGTAGGTCTCATATCACGCTGCTCCAAATTTCTTCCTCA | *BsaI* |
| GSTU27 5´ | ATGGTAGGTCTCACTCCATGTCAGAAGAAGAAGTGGTGGTG | *BsaI* |
| 3´ | ATGGTAGGTCTCATATCAATAACCATAGAAAAACTCATGGACC | *BsaI* |
| GSTU28 5´ | GGATCCATGGGGAAAGAAAATAGCAA | *Bam*HI |
| 3´ | GTCGACTCATTCAACACCAAAAATCT | *Sal*I |

**Supplementary Table 2**

Expression pattern of the genes of acrolein-compatible AtGSTU isozymes.

| isozyme | tissue distribution*^a^* | stressor to induce gene expression*^b^* |
| --- | --- | --- |
| AtGSTU2 | roots (strongly in endodermis and lateral root cap) | heat, UV-B, salt, osmotic, cold, wound |
| AtGSTU3 | sepal, adult leaf, root endodermis | UV-B, salt, oxidative, osmotic, drought, cold, wound |
| AtGSTU4 | sepal, adult leaf, senescent leaf, root endodermis | UV-B, salt, osmotic, wound |
| AtGSTU5 | roots (strongly in endodermis and lateral root cap) | heat, drought, wound |
| AtGSTU13 | various tissues except male organs | UV-B, osmotic |
| AtGSTU17 | petal, rosette leaves | cold, osmotic, wound |
| AtGSTU18 | petal, sepal, pedicel, leaves | not induced |
| AtGSTU19 | various tissues except pollen | not induced |
| AtGSTU24 | root hair zone, root endodermis, lateral root cap | heat, UV-B, oxidative, osmotic, drought, cold, wounding |
| AtGSTU28 | roots (strongly in root hair zone) | salinity, osmotic, drought |

*^a^* Extract from Fig. 3 in Dixon et al. (2010). *^b^* Extract from AtGenExpress data.
